# Gallbladder mucocele caused by intestinal metaplasia in lithiasic cholecystitis: A case report and literature review of a rare association

**DOI:** 10.1016/j.ijscr.2024.109405

**Published:** 2024-02-23

**Authors:** Marion Poget, Vilma Salvatori Chappuis, Francesc Carbó Descamps, Alend Saadi

**Affiliations:** a Children and Adolescent Surgery Service, Lausanne University Hospital, Rue du Bugnon 46, CH-1011 Lausanne, Switzerland; b ADMED Pathology, Neuchatel, Switzerland; c Surgery Department, Neuchâtel Hospital, Rue de la Maladière 45, CH-2000 Neuchâtel, Switzerland; d Obesity and Metabolic Diseases Centre, Neuchâtel Hospital, Rue de la Maladière 45, CH-2000 Neuchâtel, Switzerland; e Faculty of Biology and Medicine, University of Lausanne, Rue du Bugnon 21, CH-1011 Lausanne, Switzerland

**Keywords:** Gallbladder mucocele, Cholecystitis, Intestinal metaplasia, Carcinoma, Case report, Literature review

## Abstract

**Introduction:**

Mucin hypersecretion promoted by intestinal metaplasia can lead to gallstone formation. The presence of large amounts of mucin induced by a change in biliary epithelium structure is called a mucocele, a usually benign condition studied among animals but rarely described in humans. This entity must be distinguished from hydrops, a condition secondary to an impacted gallstone in the cystic duct leading to an outlet obstruction and distension of the gallbladder.

**Presentation of case:**

We report a case of a 51-year-old female with lithiasic cholecystitis showing areas of intestinal metaplasia associated with a mucocele. Laparoscopic cholecystectomy was performed with an uneventful postoperative course. Macroscopic findings revealed a dilated gallbladder filled with mucoid fluid. Signs of chronic and focally acute cholecystitis with areas of intestinal metaplasia were observed microscopically.

**Discussion:**

Lithiasic gallbladders can bear a gene that is found in goblet cells of intestinal metaplasia, leading to mucin hypersecretion. Metaplasia – a benign lesion often encountered on cholecystectomy specimens – can be the precursor of carcinoma. Mucin-producing gallbladder carcinoma is a very aggressive tumor that can appear as a mucocele. Consequently, preoperative computed tomography or magnetic resonance cholangiopancreatography should be performed in the presence of an unusual aspect on sonography.

**Conclusion:**

Metaplastic changes in gallbladder epithelium associated with an overproduction of mucin and lithiasic cholecystitis reported in this case is a rarity. Malignancy is an alternative diagnosis of gallbladder mucocele that must be suspected whenever preoperative imaging of the gallbladder is atypical.

## Highlights

• Alteration in mucin gene expression occurring in intestinal metaplasia promotes gallstone formation.
• Intestinal metaplasia in not an uncommon finding in gallbladders and it can be the precursor of a carcinoma.
• Abundant mucin induced by intestinal metaplasia is called a mucocele, a benign condition rarely described in humans.
• Gallbladder hydrops – secondary to an impacted gallstone in the cystic duct – is often incorrectly referred to as mucocele.
• Mucin-producing gallbladder carcinoma must be suspected whenever preoperative imaging of a gallbladder mucocele is atypical

## 1 Introduction

Glandular metaplastic changes in the gallbladder are common and associated with mucin production [1]. Three types of metaplasia of glandular epithelium of the gallbladder are encountered: intestinal, gastric, and squamous [2]. It has been reported that gastric metaplasia is the most common type of metaplasia found in the gallbladder, ranging from 3.3 % to 59.5 % of cholecystectomy specimens [[2], [3], [4], [5]], and intestinal metaplasia is found in 0.9 % to 9.8 % of cases [[2], [3], [4], [5]]. Metaplasia can further lead to dysplasia and the development of gallbladder carcinoma [2,6,7].

Gallbladder mucocele due to mucin overproduction must be distinguished from hydrops, a condition secondary to an impacted gallstone in the cystic duct leading to an outlet obstruction and distension of the gallbladder [8,9].

Mucin proteins are essential in protecting the gallbladder epithelium, preventing the bile acids from diffusing through the gallbladder wall. However, it may also promote gallstone formation when present in large amounts [10,11].

We present a case of a 51-year-old female with lithiasic cholecystitis showing areas of intestinal metaplasia associated with a mucocele. Despite the well-established correlation between mucin hypersecretion and intestinal metaplasia, this cholecystectomy specimen showed much intraluminal mucus rarely described in the literature.

## 2 Presentation of case

A 51-year-old Portuguese female presented to our regional emergency department with a complaint of right upper quadrant abdominal pain for the last two days associated with emesis but no fever. Similar intermittent pain had occurred in the postprandial phase in the past six months. She was overweight, with a BMI of 29.54 kg/m^2^, and had no past medical or surgical history. Her family medical histories do not report any relevant conditions, especially no history of hepatobiliary malignancies.

**Fig. 1:**
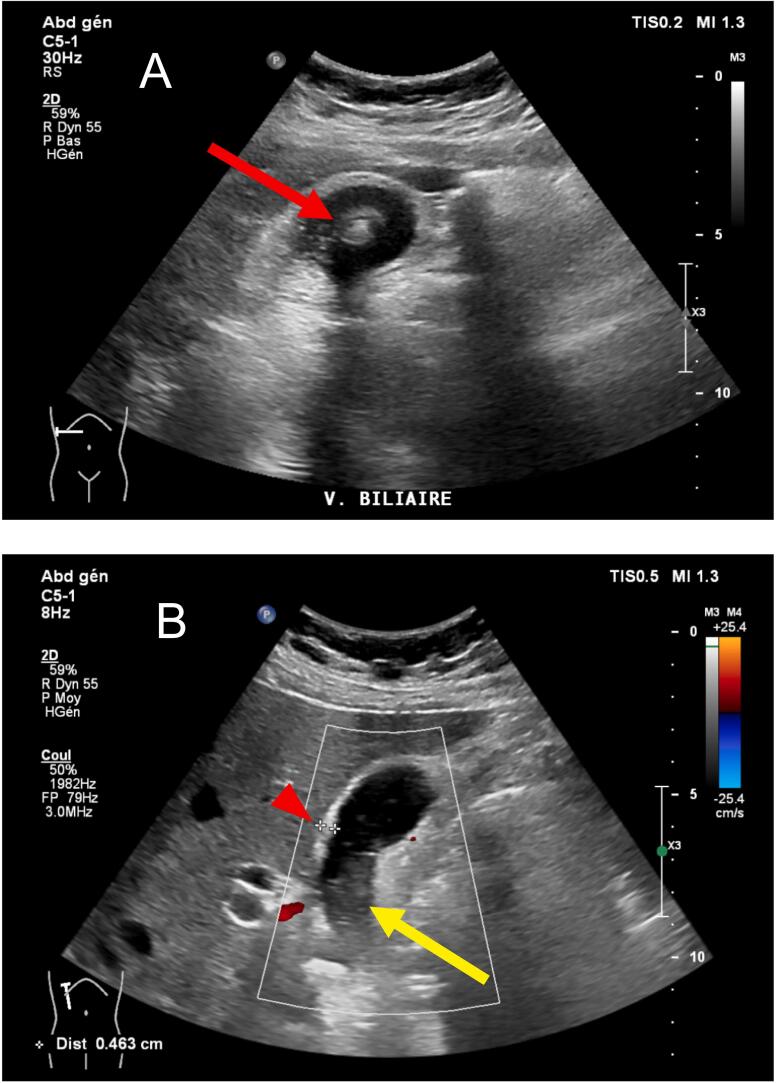
Preoperative sonography. (A) Lithiasic cholecystitis with a stone of 26 mm (red arrow). (B) Sludge (yellow arrow), thickening of the gallbladder of 4.6 mm (red arrowhead).

Upon physical examination, the patient was hemodynamically stable (pulse rate 72 beats per minute, arterial pressure 152/88 mmHg), oxygen saturation by pulse oximeter was normal (98 % in room air), and she was afebrile (36.7 °C). She had tenderness upon palpation of the right upper quadrant with a positive Murphy's sign, and the rest of the physical exam was unremarkable. Laboratory findings showed a slightly elevated leukocytosis (10.4 G/L, normal range 4.0–10.0 G/L), no elevation of C-reactive protein (CRP), and normal liver and pancreas tests. Sonography revealed signs of lithiasic cholecystitis with a stone of 26 mm, sludge, and a thickening of the gallbladder wall (Fig. 1). The patient was diagnosed with acute cholecystitis, for which an intravenous antibiotic therapy of Ceftriaxone and Metronidazole was administered before proceeding to a cholecystectomy the following day. The cholecystectomy was performed laparoscopically by a registrar surgeon using the four-port technique. The abdominal cavity exploration showed epiploic adhesions to the enlarged but otherwise normal gallbladder. The gallbladder remained intact and was then placed in a formalin solution and sent for histopathology analysis.

**Fig. 2:**
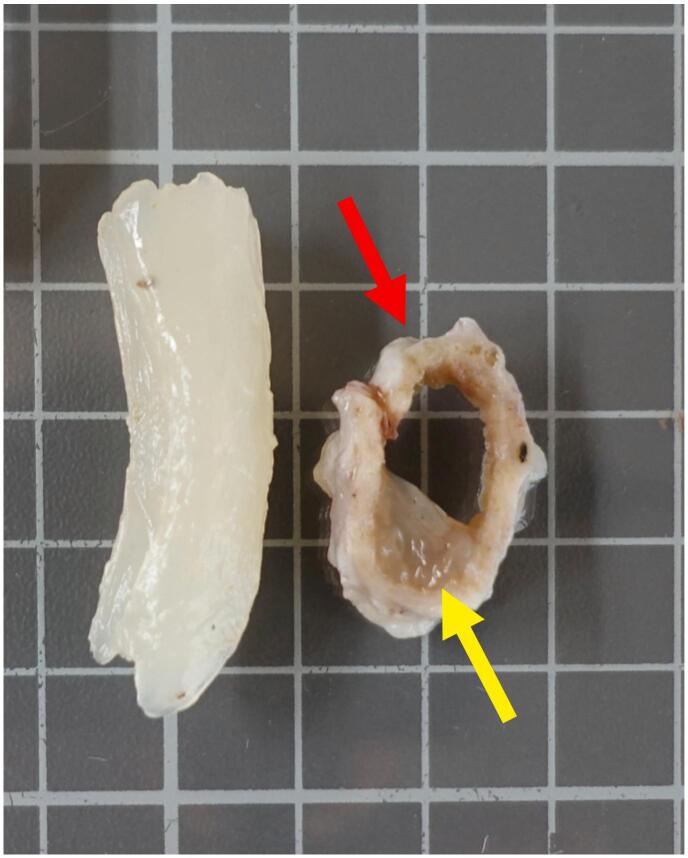
Macroscopic illustration of the gallbladder. Mucus is displayed on the left, and a transversal section of the cystic duct is shown on the right (red arrow) with adherent mucus (yellow arrow).

The macroscopic aspect of the gallbladder revealed dimensions of 11 cm long and 3.4 cm across and the external surface appeared smooth and beige/grey colored. Interestingly, the opening of the piece exposed a considerable amount of whitish mucus inside the gallbladder – a mucocele – whose mucosa appeared beige (Fig. 2). A stone impaction of 0.6 cm long in the cystic duct was noted.

**Fig. 3:**
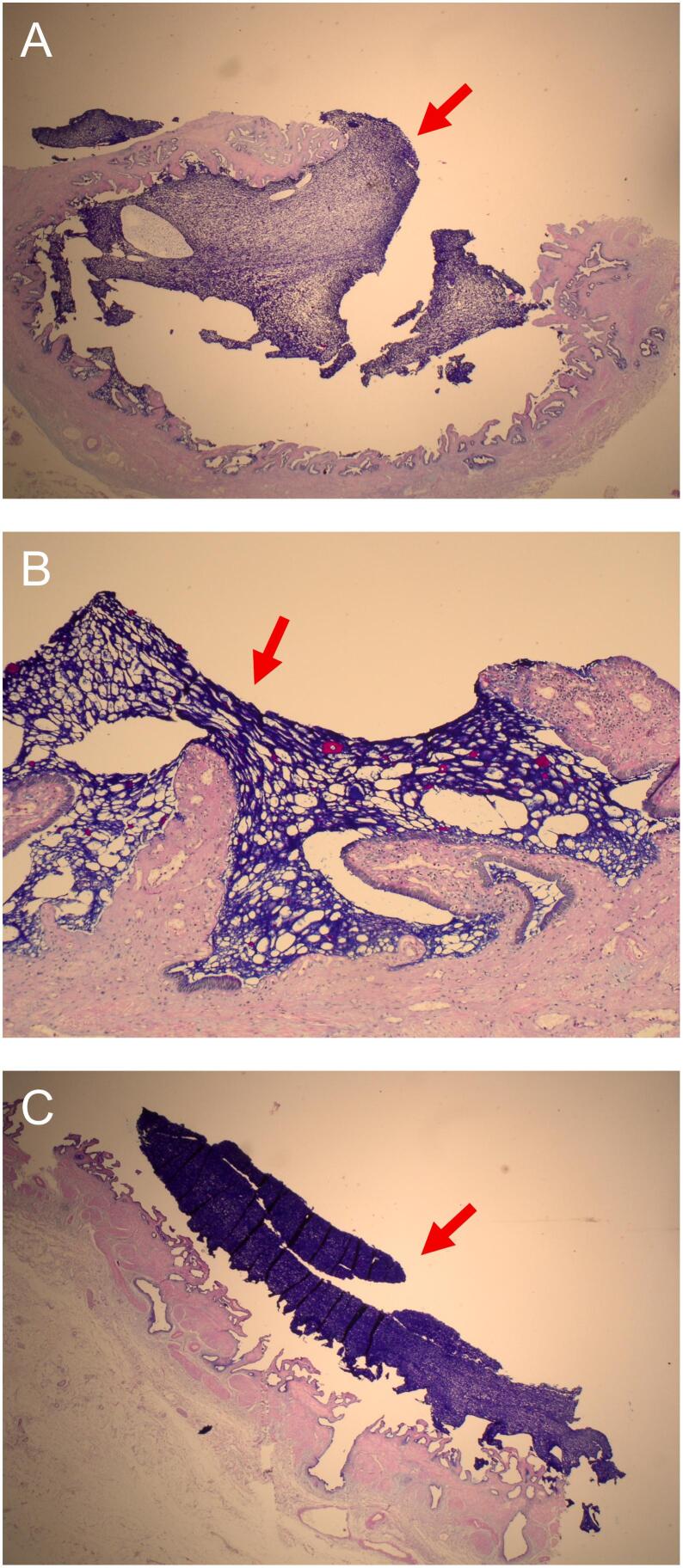
Microscopic sections showing the intraluminal mucus. (A), (B) Mucus (red arrow) adheres to the normal biliary epithelium. Alcian blue-Periodic acid-Schiff (AB-PAS), magnification x 40 and × 200. (C) Intraluminal mucus, magnification x 40.

**Fig. 4:**
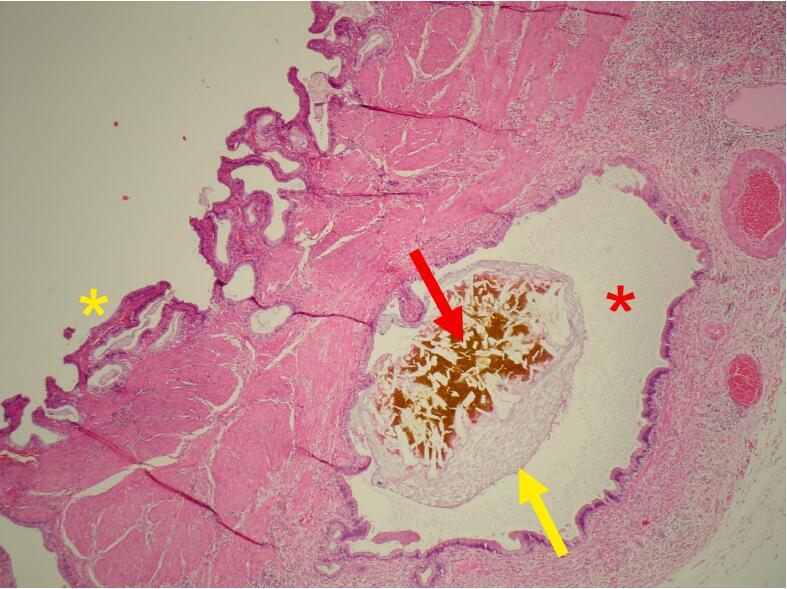
Microscopic illustration of the gallbladder. A Rokitansky-Aschoff sinus (red asterisk) contains a stone fragment (red arrow) and mucus (yellow arrow). The normal biliary epithelium is marked with the yellow asterisk. Haematoxylin and eosin, magnification x 40.

**Fig. 5:**
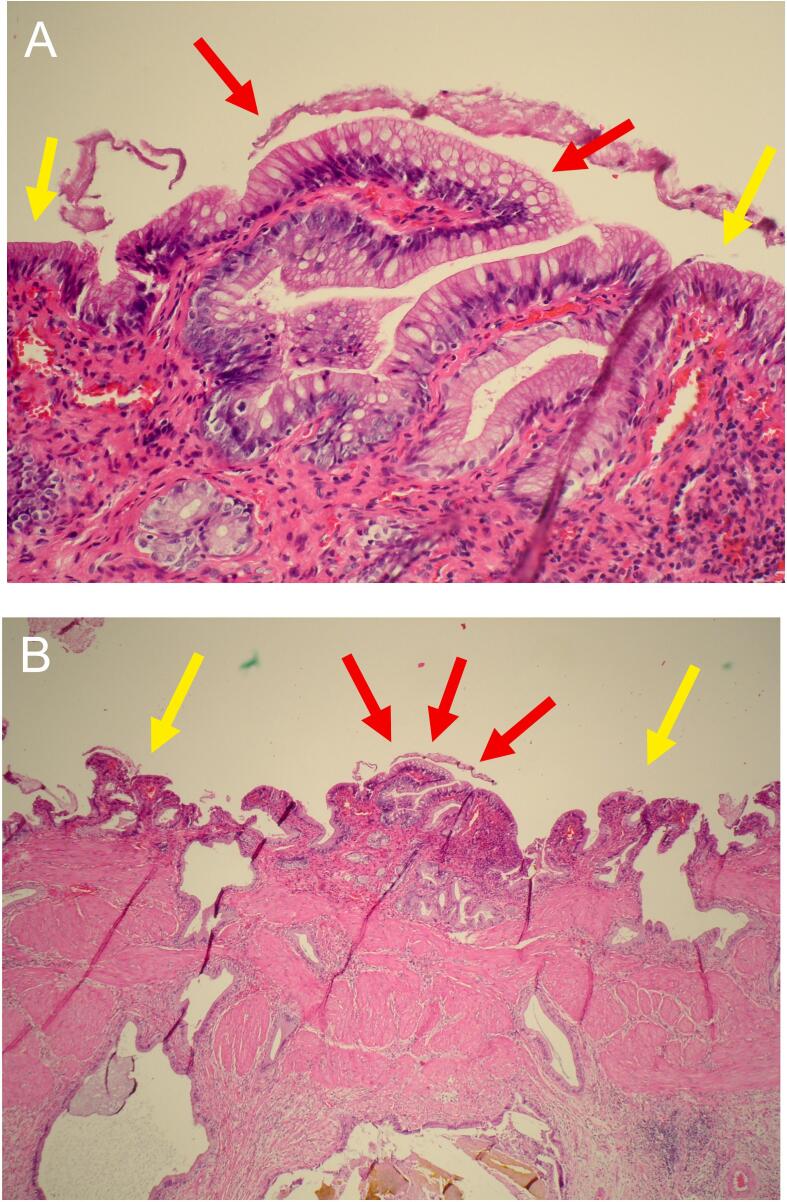
Microscopic illustration of the gallbladder. (A) Intestinal metaplasia (red arrow) without dysplasia. Normal biliary epithelium (yellow arrow). Haematoxylin and eosin, magnification x 40. (B) The same section at magnification x 200. A single layer of columnar cells with basally located nuclei is seen in the normal biliary epithelium. Intestinal metaplasia is characterized by the presence of goblet cells, usually found in the small intestine epithelium.

The microscopic examination revealed abundant intraluminal mucus (Fig. 3) along with chronic cholecystitis with focal acute inflammation with dilated Rokitansky-Aschoff sinuses containing lithiasic fragments and mucus (Fig. 4). A few areas of mucus-secreting intestinal metaplasia were observed with no signs of dysplasia (Fig. 5).

The immediate postoperative course was uneventful, and the patient was discharged the day following surgery. No significant complaint was reported at the one-month follow-up, and the physical examination revealed unremarkable findings.

This article has been reported in line with the SCARE criteria [12].

## 3 Discussion

We report a case of gallbladder mucocele associated with intestinal metaplasia in lithiasic cholecystitis, a rarely described pathological finding.

A literature review was performed using PubMed database using the keywords “gallbladder”, “mucocele”, “mucin”, “metaplasia” and “carcinoma”, which generated 249 articles. We included case reports, case series, and original articles, and we excluded review articles, editorials, and irrelevant articles based on the title or the abstract. We assessed 111 articles for eligibility; eight were excluded for insufficient patient information. A total of 103 articles on gallbladder mucocele – either of benign or malignant origin – were included, with a majority of animal-related articles. Mucocele associated explicitly with intestinal metaplasia was found in only one article (a case report).

Gallbladder mucocele has been extensively studied among animals, especially dogs [[13], [14], [15]], but rarely described in humans [8]. The literature review revealed only one case report of a mucocele induced by intestinal metaplasia in a human cholecystectomy specimen by Clemente et al. [8]. They reported the case of a 53-year-old female with a 1-year history of right upper quadrant abdominal pain and a 4-cm polyp of the gallbladder protruding into the lumen on the abdominal ultrasound and computerized tomography. Laboratory findings were not indicated. The patient underwent laparoscopic cholecystectomy, and the polypoid lesion found upon opening of the gallbladder was covered by macroscopically normal mucosa. No gallstone was identified in the gallbladder lumen. Upon histopathology, the dissection of the polypoid lesion revealed a collection of whitish mucus with intracavitary intestinal mucosa.

Gallbladder mucocele in dogs has been widely described and can be a life-threatening condition due to the high risk of gallbladder rupture [16,17]. The etiopathogeny of this condition remains unclear, but it is thought that mucinous hyperplasia leads to an accumulation of intraluminal thickened bile [17]. The large amount of intraluminal inspissated bile leads to gallbladder distension, which can further result in transmural necrosis and gallbladder rupture [16]. The etiology of mucocele formation in dogs is unknown; whether bacterial growth plays a role in the pathogenesis of gallbladder mucocele remains unclear [13,14]. Certain endocrinopathies, such as hyperadrenocorticism and hypothyroidism, are believed to be associated with the development of gallbladder mucocele in dogs [15]. Diagnosis of mucoceles in dogs can be achieved by preoperative sonography, but histopathology analysis makes the definitive diagnosis. Malek et al. [18] report that 74 % of gallbladder mucoceles were preoperatively diagnosed with sonography. Ultrasonographic features that may hint at the diagnosis of gallbladder mucocele are based on the presence of an immobile, stellate, or finely striated bile pattern [17]. These latter features contrast with our imaging finding of intraluminal biliary sludge or the visualization of a polyp in the case presented by Clemente et al. [8]. Macroscopically, gallbladder mucoceles in dogs are described as shiny, gelatinous, greenish-black, sometimes with a lamellar or striated pattern, which differs from our findings and Clemente's [8]. Upon histopathology findings, cholecystitis is found in 9.3 % to 28.8 % of dogs who undergo cholecystectomy for a gallbladder mucocele [16,18,19], and no evidence of gallstones is mentioned in these studies. Interestingly, veterinarians do not make the confusion between mucocele and hydrops since mucocele is a condition frequently encountered in their practice. This present article aims to clarify this uncommon situation in humans.

Mucin proteins play a significant role in the protection of the gallbladder epithelium. However, they also form cholesterol and pigment gallstones when mucin hypersecretion occurs.

Consequently, intestinal metaplasia has been reported to be associated with cholelithiasis [11]. Human mucin genes have been identified and studied in healthy and pathological gallbladders. MUC1, MUC3, and MUC4 are called membrane-bound mucins, and MUC2, MUC5AC, MUC5B, and MUC6 are called secretory mucins [11,20]. Lee et al. [20] found that lithiasic gallbladders showed an altered mucin gene expression, with the presence of MUC2 and MUC4 and an increase in MUC1, MUC3, MUC5B, and MUC6 expression. They suggest that the higher the expression of MUC2, MUC3, MUC5B, and MUC6, the more significant the amount of mucin in gallbladders. Goblet cells of the small bowel and the colon have been reported to express MUC2 [21], and Lee et al. [20], found that goblet cells in intestinal metaplasia of the gallbladders expressed MCU2. MUC2 was absent in healthy gallbladder specimens, similar to what Ho et al. [11] described.

Metaplasia is a common finding in cholecystectomy specimens [[2], [3], [4], [5]], and it can be a precursor of dysplasia, which can progress to gallbladder carcinoma [2,6,7]. The worldwide age-standardized incidence rate of gallbladder cancer was 1.2 per 100.000 standard population in 2020, and it is the 23rd most common cancer worldwide [22]. Gallbladder carcinomas can be incidentally detected on cholecystectomy specimens, with a reported prevalence ranging from 0.78 % to 2.49 % [2,4,23]. If symptomatic, patients present with nonspecific, cholecystitis-like symptoms such as pain, nausea, anorexia, and sometimes obstructive jaundice [24,25]. Most gallbladder carcinomas can produce mucin, but mucin-producing carcinomas with clinical significance due to the abundance of mucin are extremely rare [24,26]. These mucin-producing gallbladder carcinomas (MPGBC) encompass two different types of carcinomas: well-differentiated adenocarcinoma that displays a papillary growth pattern and mucinous carcinoma [24]. These MPGBC account for 2.5 %–5.5 % of all gallbladder carcinomas and are more aggressive than the others [25]. The sonography of MPGBC typically reveals linear hyperechoic contents or an intraluminal polypoid/irregular mass [24,26,27], but malignancy is best evaluated on magnetic resonance cholangiopancreatography (MRCP) or computed tomography [26]. MRCP of MPGBC typically shows linear or curvilinear striations in the gallbladder called the “mucous thread” sign by Ishiguro et al. [26] or a stellate soft tissue mass in the gallbladder [27]. These images of striations are thought to arise from the highly viscous properties of the mucin [26]. In our case, the patient's sonography did not display the typical linear hyperechoic contents. Hence, the suspicion of a malignancy was not raised.

Unfortunately, a photograph of the fresh gallbladder displaying the large amount of mucin is lacking; a formalin-fixed specimen alone is available. The impacted gallstone in the cystic duct may be a confounding factor for the presence of abundant mucin. However, the association with intestinal metaplasia remains a rare entity worth reporting. A genetic analysis of the mucin gene expression in our patient would have been valuable to evaluate whether the mucin expression status correlates with the present literature review. Unfortunately, the genetic analysis was not performed for two reasons: the costs would have been on the patient's behalf if this additional exam had been performed as soon as the pathological findings were available, given the unnecessity of obtaining a genetic profile to provide adequate care for the patient. Furthermore, the institute of pathology did not possess the sample anymore at the time of the article's writing.

To the best of our knowledge, our case of lithiasic cholecystitis associated with gallbladder mucocele secondary to intestinal metaplasia is the second case described in the literature after Clemente et al. [8]. The management of this condition is equivalent to lithiasic cholecystitis with hydrops; both require a laparoscopic cholecystectomy. Gallbladder mucocele secondary to intestinal metaplasia is a fortuitous discovery and is a benign condition requiring no further follow-up. However, malignancy should be suspected in the case of an unusual sonographic aspect for cholelithiasis (irregular/polypoid mass, striations) requiring an MRCP before surgery.

## 4 Conclusion

Metaplastic changes in gallbladder epithelium are common, but these changes associated with an overproduction of mucin – a mucocele – and lithiasic cholecystitis reported in this case are a rarity. Furthermore, this present article aims to clarify the difference between mucocele and hydrops, often incorrectly referred to as the same entity in the literature. Finally, malignancy is an alternative diagnosis of gallbladder mucocele that must be suspected whenever preoperative imaging of the gallbladder is atypical, which advocates the need for an MRCP prior to surgery.

## Consent

Written informed consent was obtained from the patient involved in this case report.

## Ethical approval

Ethical approval not required.

## Funding

This research did not receive any specific grant from funding agencies in the public, commercial, or not-for-profit sectors.

## Author contribution

Dr. Marion Poget contributed to the writing and submission of the case report, and to the operation of the patient who was the subject of this case report. Dr. Alend Saadi contributed to the editing of the article. Dr. Vilma Salvatori Chappuis and Dr. Francesc Carbó Descamps contributed to the pathology findings, report, and images.

All authors contributed to the article and approved the submitted version.

## Guarantor

Dr Alend Saadi.

Dr Marion Poget.

## Research registration number

This project is not a research study and therefore does not need registration.

## Declaration of generative AI and AI-assisted technologies in the writing process

During the preparation of this work, the authors used Grammarly, Inc. in order to improve readability and language. After using this tool, the authors reviewed and edited the content as needed and take full responsibility for the content of the publication.

## Declaration of competing interest

The authors have no conflicts of interest to disclose.
